# Thin silica shell coated Ag assembled nanostructures for expanding generality of SERS analytes

**DOI:** 10.1371/journal.pone.0178651

**Published:** 2017-06-01

**Authors:** Myeong Geun Cha, Hyung-Mo Kim, Yoo-Lee Kang, Minwoo Lee, Homan Kang, Jaehi Kim, Xuan-Hung Pham, Tae Han Kim, Eunil Hahm, Yoon-Sik Lee, Dae Hong Jeong, Bong-Hyun Jun

**Affiliations:** 1 Department of Chemistry Education, Seoul National University, Seoul, Republic of Korea; 2 Department of Bioscience and Biotechnology, Konkuk University, Seoul, Republic of Korea; 3 Interdisciplinary Program in Nano-Science and Technology. Seoul National University, Seoul, Republic of Korea; 4 School of Chemical and Biological Engineering, Seoul National University Seoul, Republic of Korea; Institute of Materials Science, GERMANY

## Abstract

Surface-enhanced Raman scattering (SERS) provides a unique non-destructive spectroscopic fingerprint for chemical detection. However, intrinsic differences in affinity of analyte molecules to metal surface hinder SERS as a universal quantitative detection tool for various analyte molecules simultaneously. This must be overcome while keeping close proximity of analyte molecules to the metal surface. Moreover, assembled metal nanoparticles (NPs) structures might be beneficial for sensitive and reliable detection of chemicals than single NP structures. For this purpose, here we introduce thin silica-coated and assembled Ag NPs (SiO_2_@Ag@SiO_2_ NPs) for simultaneous and quantitative detection of chemicals that have different intrinsic affinities to silver metal. These SiO_2_@Ag@SiO_2_ NPs could detect each SERS peak of aniline or 4-aminothiophenol (4-ATP) from the mixture with limits of detection (LOD) of 93 ppm and 54 ppb, respectively. E-field distribution based on interparticle distance was simulated using discrete dipole approximation (DDA) calculation to gain insight into enhanced scattering of these thin silica coated Ag NP assemblies. These NPs were successfully applied to detect aniline in river water and tap water. Results suggest that SiO_2_@Ag@SiO_2_ NP-based SERS detection systems can be used as a simple and universal detection tool for environment pollutants and food safety.

## Introduction

Surface-enhanced Raman scattering (SERS) is a sensitive optical detection tool. It is popular for identifying and detecting chemical and biological species due to its single molecular sensitivity and non-destructive feature [1–6]. Therefore, many researchers have extensively explored direct and label-free SERS detection strategies based on distinguished SERS peaks of molecules in biomedicine field for multiplex high-throughput screening and pollutant monitoring [7–10].

When SERS technique is used for chemical detection, only target molecules located close to the metal surface can be analyzed [11–13]. However, the observed SERS intensity is affected by the affinity of functional group to the metal surface rather than by the quantity of targets. For instance, molecules with stronger metal affinity such as thiol group-containing molecules can be adsorbed more strongly to the metal surface than those with weaker metal affinity, thus limiting simultaneous and quantitative detection of analytes with different metal-affinities. This remains a challenge to achieve universal and direct detection using SERS technique.

Metal nanostructures play important roles in generating SERS signals [14,15]. Up to date, single nanoparticle (NP) based structures such as gold NPs, silver NPs, and other metal-based structures have been widely used [16–29]. However, single NPs hinder broad application due to their low SERS activities [14,30,31]. To overcome this problem, aggregated Au or Ag NPs structures and anisotropic NPs such as gold nanochain and gold nanostar have been introduced to further enhance SERS signals [14,32–35]. they have also been used to detect various molecules [36–39]. Nanostructure containing Ag NPs on a silica NP core can produce intense SERS signals with a narrow intensity distribution for immunoassay and multiplexed bio-molecule detection [39–43].

Recently, Au NPs coated with thin and optically transparent silica shell have been used for food safety assessments and detecting environmental pollutants [44,45]. Although thin silica shell coated Au NPs have been used as SERS probes for detecting such chemicals, the problem of affinity differences of functional groups to the metal surface has not been solved. In addition, single Au NPs hinder broad application due to their low SERS sensitivity.

Assembled Au or Ag NPs with thin silica coating might exhibit intense SERS activity due to their many hot spots caused by assembled structure. Using a thin silica shell might solve the problem of affinity differences of various functional groups to the metal surface [14,31].

Recently, our group developed a nanostructure by assembling Ag NPs onto surface of silica NP. These structures have intense SERS activity due to their many hot spots, they have utilized highly sensitive SERS nanoprobe with several advantages such as easy handling and reproducibility of preparation [5,6,46]. Due to its sensitivity (up to single particle SERS measurement), the problem of affinity differences of various functional groups to the metal surface is solved. It can be used to detect various kinds of SERS signals of small molecules.

Here, we introduce thin silica shell coated Ag for assembled silica NPs (SiO_2_@Ag@SiO_2_ NPs). These SiO_2_@Ag@SiO_2_ NPs can generate several higher orders of magnitude in intensity of Raman scattering between hot junctions of Ag NPs in thin silica shell and Raman label compounds adjacent to the shell. These SiO_2_@Ag@SiO_2_ NPs could simultaneously detect a mixture of aniline (amine group containing chemical) and 4-aminothiophenol (thiol group containing chemical) by SERS. Limits of detection (LOD) for aniline and 4-aminothiophenol (4-ATP) were compared to test the feasibility of having a universal SERS substrate to quantify analytes. Optical properties of this structure were characterized using theoretical discrete dipole approximation (DDA) calculations. Their practical feasibility for detecting aniline in river water and tap water was also determined.

## Experimental details

### Chemicals and materials

Tetraethylorthosilicate (TEOS), 3-mercaptopropyl trimethoxysilane (MPTS), ethylene glycol, silver nitrate (AgNO_3_, 99.999%), octylamine, 4-aminothiophenol (4-ATP), and aniline were purchased from Sigma-Aldrich (St. Louis, MO, USA). Absolute ethanol (99.8%), ammonium hydroxide (NH_4_OH, 27%), oleic acid, and ethanol (98%) were purchased from Daejung (Siheung, Korea).

### Preparation of Ag NPs assembled silica NPs

TEOS (1.6 mL) was dissolved in 40 mL of absolute ethanol containing 3 mL NH_4_OH and stirred vigorously at 25°C for 20 h. Resulting silica NPs were centrifuged at 8,500 rpm for 15 min, and washed with ethanol several times to remove the excess reagent. These silica NPs were functionalized with a thiol group. Briefly, 200 mg of silica NPs, 200 μL MPTS and 40 μL NH_4_OH were dispersed in 8 mL of ethanol. After the mixture was stirred at 25°C for 6 h, these MPTS-treated silica NPs were centrifuged at 8,500 rpm for 15 min, and washed several times with ethanol. After that, 25 mL aliquot of AgNO_3_ solution in ethylene glycol was mixed with MPTS-treated silica NPs thoroughly. Then 41.24 μL of 5 mM octylamine was added rapidly to this solution. The resulting dispersion was stirred at 25°C for 1 h. These silica Ag NPs were then centrifuged at 8,500 rpm for 15 min, and washed several times with ethanol. Oleic acid (1 mM) was then mixed with 10 mg of Ag NPs with stirring at 25°C overnight. The resulting surface modified NPs were recovered by centrifugation (13,000 rpm for 10 min) and washed several times with ethanol.

### Thin silica coating of Ag NPs assembled silica NPs

MPTS (20 mM) and 3.71 μL NH_4_OH (27%) were added to the reaction mixture under vigorous stirring followed by shaking while adding TEOS (1:25 dilution with 27% NH_4_OH). The mixture was allowed to react at 25°C for 20 h. The prepared thin silica coating of Ag NPs assembled silica NPs were then centrifuged at 8,500 rpm for 15 min, and washed with ethanol five times, and re-dispersed in ethanol.

### Raman instrument and measurements

Raman measurements were conducted using a confocal micro Raman system (LabRam 300, JY-Horiba) equipped with an optical microscope (Olympus, Tokyo, Japan). Raman scattered signals were collected in a back-scattering geometry and detected using a spectrometer equipped with a thermoelectrically cooled (−70°C) CCD detector. The excitation laser was focused. Raman signals were collected using a 10× objective lens (NA 0.25, Olympus). LOD of the thin shell NP system for detecting aniline was calculated based on standard deviation (SD) of the response and the slope of the calibration curve (S) at levels approximating the LOD [LOD = (SD/S)] using a laser beam with diameter of 2.6 μm. SiO_2_@Ag@SiO_2_ NPs were dispersed in aniline mixed 4-ATP in deionized water with EtOH for SERS measurements. 4-ATP was dispersed in water with pH 8. This was mainly caused by a dilution effect and the addition of HCl or NaOH. After 15 min of shaking, SiO_2_@Ag@SiO_2_ NPs were injected into a capillary tube. A similar process was applied to measuring LODs of aniline, 4-ATP, and aniline in river water and tap water. SERS spectra of all samples were measured using ×10 objective lens (NA 0.25) with photoexcitation at 532 nm, laser power of 10 mW, and acquisition time of 10 s.

### Theoretical E-field calculation

E-fields of SiO_2_ encapsulated Ag NP monomer and Ag NP dimers with different inter-particle distances were calculated using DDA (DDSCAT 7.1) to support SERS enhancement of SiO_2_@Ag@SiO_2_ NPs [47]. Each dimension of the calculated structure was obtained from HR-TEM analysis. The diameter of an Ag NP was 16 nm. The thickness of the SiO_2_ shell was measured at 3 nm. The medium surrounding calculated structures was set to vacuum with a refractive index of 1.00+0i. The irradiated wavelength was at 532 nm.

## Results and discussion

### Preparation of thin shell coated Ag NPs

**Fig 1:**
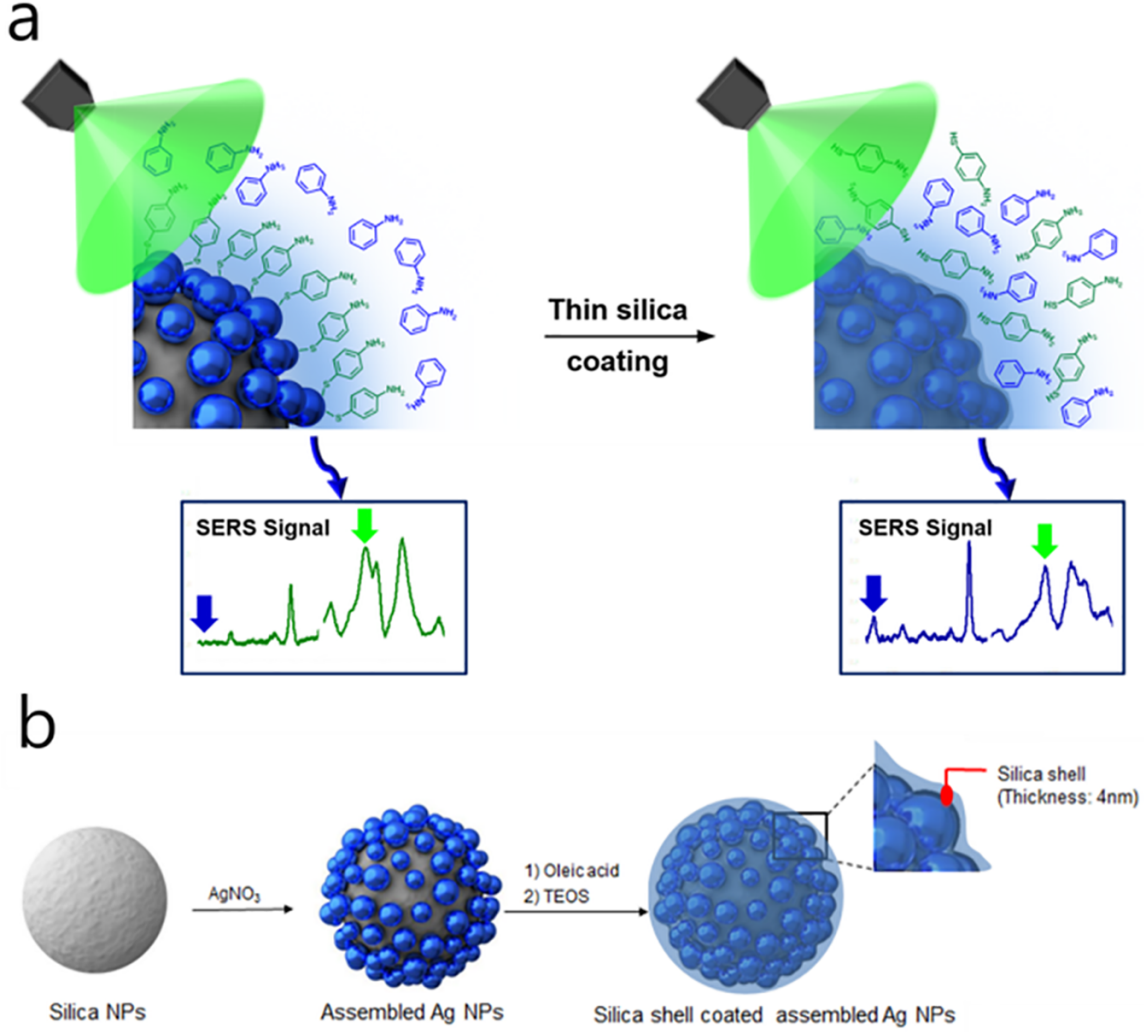
Schematic illustration of detection concept and fabrication process. (a) Simultaneous quantitative detection of aniline and 4-aminothiophenol with thin silica shell coated Ag NP assembled structure (SiO_2_@Ag@SiO_2_ NP), (b) Overall fabrication scheme of desired structure.

**Fig 2:**
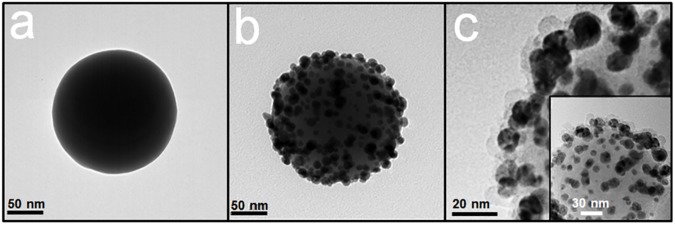
Transmission electron microscopic images of fabricated nanostructure. (a) Silica nanoparticle (NP), (b) SiO_2_@Ag NPs, and (c) SiO_2_@Ag@SiO_2_ NPs.

Fig 1 shows the fabrication flow for the proposed thin shell coated Ag NPs assembled on silica cores (SiO_2_@Ag@SiO_2_ NPs). These SiO_2_@Ag@SiO_2_ NPs had 4–5 nm of silica shell on Ag NPs assembled silica cores. Briefly, 180 nm silica core NPs were prepared using the Stӧber method (Fig 2A) [48–50]. These silica particles were functionalized with 3-mercaptopropyl-trimethoxysilane (MPTS) to introduce thiol group with high affinity for Ag NPs. These Ag NPs were then introduced onto thiol-modified silica core NPs using the modified polyol method [51–53]. Silver nitrate was dissolved in ethylene glycol as a reducing agent and solvent. They were then mixed with MPTS-treated silica NPs dispersion. After stirring, Ag NPs with diameter of 10 nm formed on the surface of silica cores (SiO_2_@Ag NPs; Fig 1B). Oleic acid was then added to these Ag NPs for fine control of the silica coating step to fabricate thin silica shell on the surface of these Ag NPs. A 4–5 nm of thin silica shell formed on these Ag NPs after adding MPTS and tetraethylorthosilicate (TEOS) to NPs dispersion under vigorous stirring (Fig 2C).

### Comparison of silica coated SiO_2_@Ag NPs and SiO_2_@Ag NPs without silica coating

Occasionally, SERS signal can be affected by the affinity of an analyte functional group for the metal surface rather than by the quantity of targets [54,55]. In particular, metal has a strong affinity for thiol groups. Thus, available target molecules for SERS detection are highly limited. Aniline is widely used in dye, drug, and organic chemical industries. The toxicity of aniline is generally associated with methemoglobin formation and damage to red blood cells [56–60]. Aniline was chosen in this study as a non-thiol-containing model toxic chemical to show that these SiO_2_@Ag@SiO_2_ NPs could be used in broader applications using SERS for label-free detection. SiO_2_@Ag NPs and SiO_2_@Ag@SiO_2_ NPs were compared using aniline and 4-ATP containing thiol moiety.

**Fig 3:**
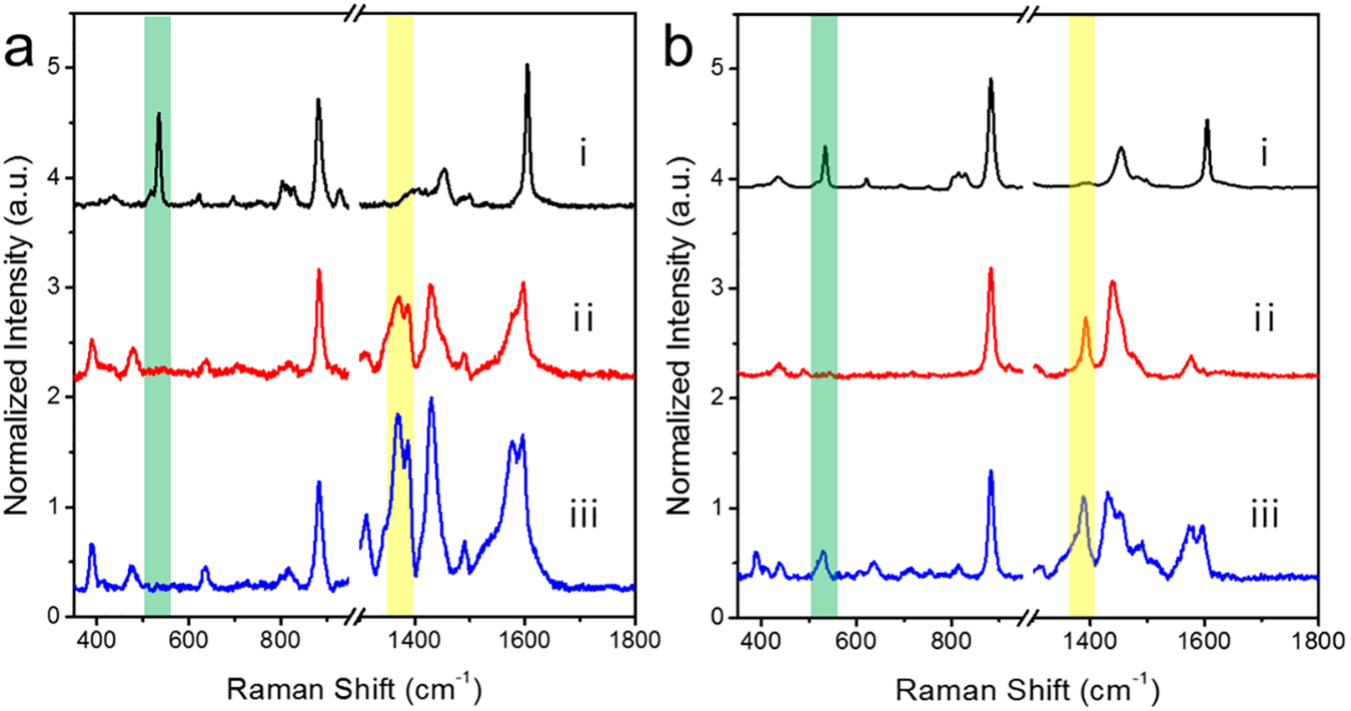
Comparison of surface-enhanced Raman scattering (SERS) spectra of a non-thiol analyte (i; aniline), a thiol analyte (ii; 4-ATP) and their mixture (iii; same quantities of aniline and 4-ATP). (a) Raman spectra of SiO_2_@Ag NPs and (b) SiO_2_@Ag@SiO_2_ NPs. Raman spectra were obtained by 532 nm photoexcitation and 10s acquisition. Intensities were normalized to Raman intensity of the ethanol peak at 882 cm^−1^. The characteristic aniline peaks were not detected in the spectrum of the mixture when the SiO_2_@Ag NP SERS substrate was used. However, both peaks of aniline and 4-ATP were detected at similar intensities when the SiO_2_@Ag@SiO_2_ NPs was used as SERS substrate. Baselines were adjusted for the clarity of comparison.

SiO_2_@Ag NPs and SiO_2_@Ag@SiO_2_ NPs were dispersed in 4-ATP or aniline solution. Both NPs exhibited unique aniline and 4-ATP SERS peaks at 520 and 1,390 cm^−1^, respectively (Fig 3A(i), 3A(ii), 3B(i) and 3B(ii)). This result indicates that SiO_2_@Ag@SiO_2_ NPs with a thin silica shell can be used for SERS-based chemical detection. In general, molecules with thiol-group have stronger affinity to the surface of gold and silver nanostructures than molecules with other functional groups such as amine. Therefore, in a mixture of various molecules, SERS intensity is dominated by molecules with thiol-groups. This phenomenon will reduce the generality of SERS detection, even though it is highly sensitive to single molecule level detection. However, such affinity propensity can be overcome by using thin shell of silica on SERS active surface. As shown in Fig 1, localized optical field was still strong at the surface of silica shell with thickness of 2–3 nm from the metal surface. Such surface prevented predominant interaction of thiol-groups with the surface, allowing other molecules such as aniline to be adsorbed onto silica surface. Their SERS intensities and thiolated molecules can be quantitatively measured. Ag NPs on a silica sphere have strong affinity for the thiol group in 4-ATP. Therefore, 4-ATP is dominantly adsorbed onto the Ag surface [61–63]. On the other hand, when a mixture of aniline and 4-ATP was added to SiO_2_@Ag@SiO_2_ NPs, 4-ATP and aniline SERS bands showed similar intensities (Fig 3B(iii)), indicating that both chemicals could be detected by SERS regardless of thiol content. Therefore, such SiO_2_@Ag@SiO_2_ SERS substrate can detect analytes regardless of their silver affinities.

### Detection of chemicals at various concentrations by SiO_2_@Ag@SiO_2_ NPs

**Fig 4:**
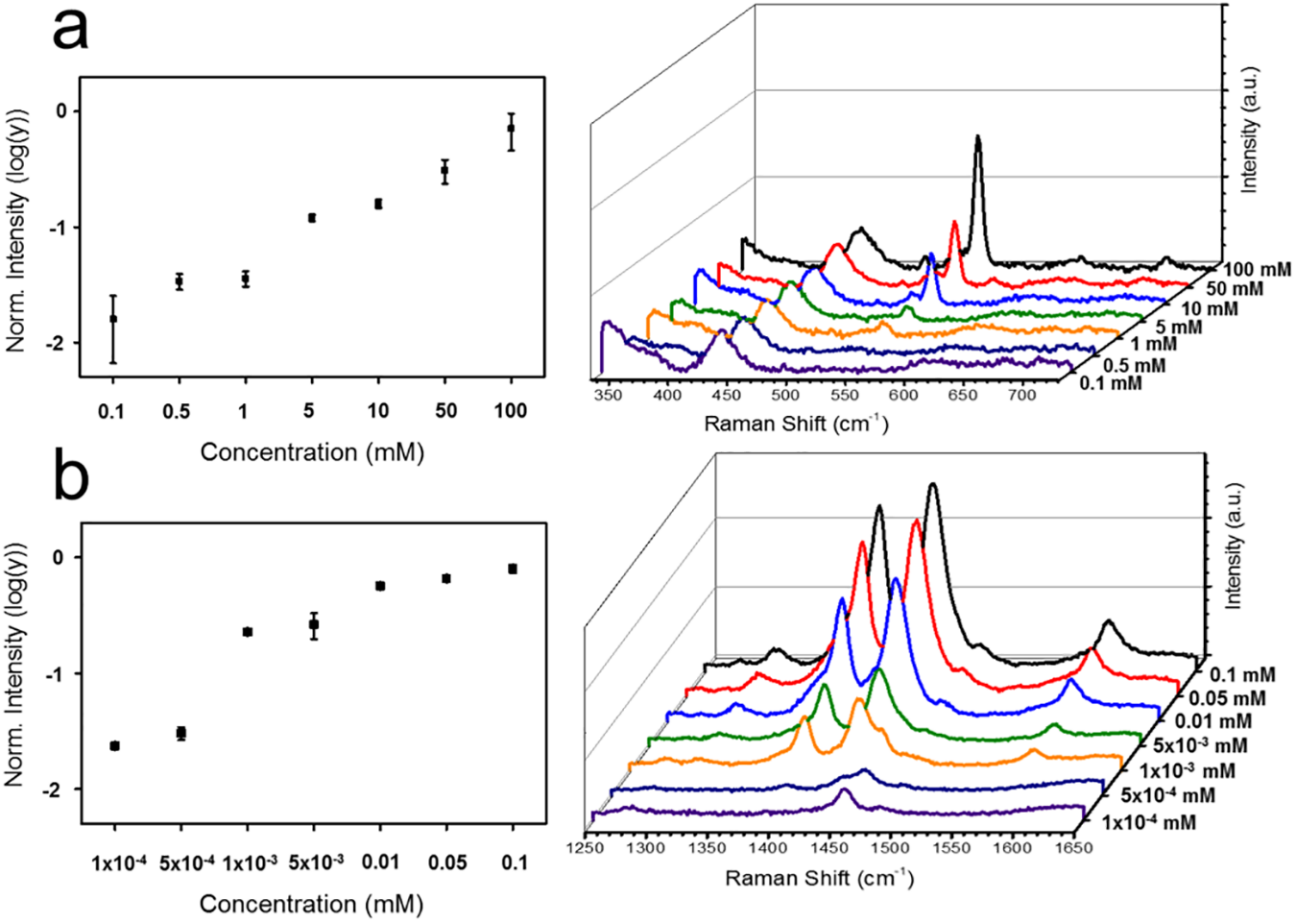
Limit of detection analysis with two different molecule. Limit of detection of (a) aniline, (b) 4-ATP at various concentrations based on their corresponding surface-enhanced Raman scattering (SERS) signals using SiO_2_@Ag@SiO_2_ NPs. All Raman spectra were measured at laser power of 10 mW with acquisition time of 10 s. Intensities were normalized to Raman intensity of ethanol peak at 882 cm^−1^.

A control experiment was performed to demonstrate the sensitivity of thin shell-coated Ag NPs based SERS detection probe. SiO_2_@Ag@SiO_2_ NPs were added to ethanol solution containing various concentrations of aniline (0.1–100 mM). Fig 4A shows SERS signals of aniline at each concentration. It illustrates that these SiO_2_@Ag@SiO_2_ NPs are active SERS substrates suitable for quantifying analytes. Using the intensity at 520 cm^−1^ for Raman band characteristic of aniline, the LOD of SiO_2_@Ag@SiO_2_ NPs for aniline was found to be 93 ppm. Its dynamic range of detection was increased to 100 mM.

Fig 4B shows 4-ATP SERS signals at various concentrations (0.1 nM–0.1 mM). Using the intensity of 1390 cm^−1^ Raman band characteristic of 4-ATP, the LOD of SiO_2_@Ag@SiO_2_ NPs for 4-ATP was found to be 54 ppb. Its dynamic range of detection was increased to 0.1 mM. These results suggest that these SiO_2_@Ag@SiO_2_ NPs can be utilized for SERS detection of these chemicals.

### Simulation of Raman signal enhancement by SiO_2_@Ag@SiO_2_ NPs

**Fig 5:**
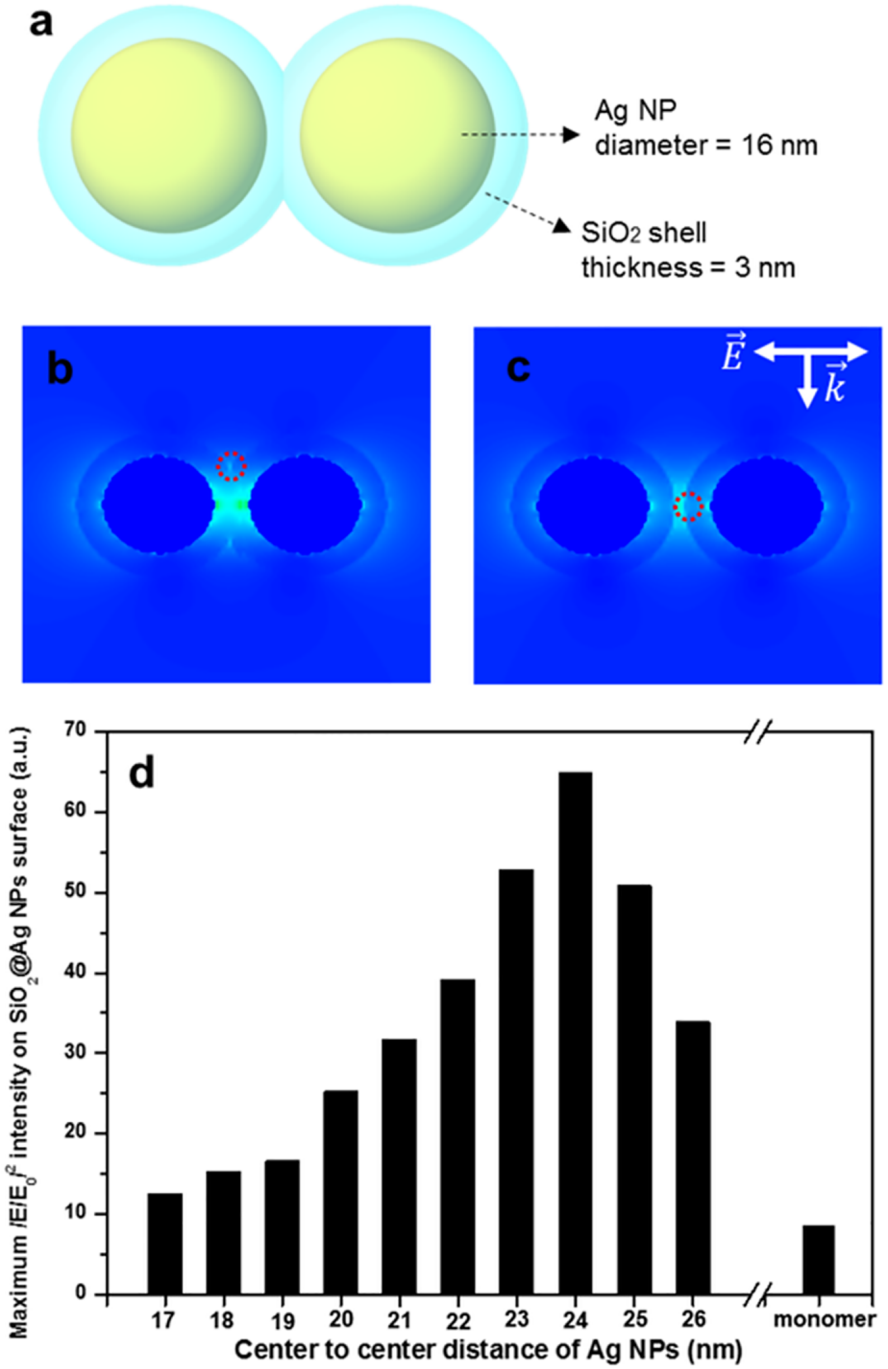
Theoretical simulation of optical fields for SiO_2_@Ag NPs with different inter-particle distance. **The wavelength of incident light was at 532 nm.** (a) An illustrated model for the nanostructure used for calculation. (b) An E-field distribution map around the nanostructure when the center-to-center distance of two spheres is smaller than the outer diameter of a sphere. The area depicted in red circle is the brightest area on silica shell surface. (c) The same as in (b) when the center-to-center distance of two spheres is larger than the outer diameter of a sphere. The area depicted in red circle is the brightest area on silica shell surface. The area depicted in red circle of (b) and (c) is the maximum (E/E_0_)^2^ of silica shell surface and same region of interest (d). A plot of (E/E_0_)^2^ at the brightest spot on silica shell surface versus the center-to-center distance of Ag NPs.

To gain insight into SERS enhancement of SiO_2_@Ag@SiO_2_ NPs, theoretical E-field distribution of SiO_2_@Ag NP monomer and dimers with different inter-particle distance was calculated by discrete dipole approximation (DDA). E-field distribution maps around the nanostructure and a plot of (E/E_0_)^2^ at the brightest spot on silica shell surface vs the center-to-center distance of Ag NPs are shown in Fig 5. As shown in the HR-TEM image (Fig 2C), the measured SERS intensity of SiO_2_@Ag@SiO_2_ NPs was an ensemble average of enhanced Raman scattered light by numerous Ag NP monomers and dimers on these SiO_2_@Ag@SiO_2_ NPs. As Ag NPs formed dimers, E-field intensity of dimers was increased compared to that of monomer due to E-field concentration between Ag NPs. Furthermore, the E-field intensity of Ag NP dimers was the highest when the distance between Ag NPs was at 8 nm. In this case, we took the value of optical field at the surface of silica shell rather than the center of inter-particle axis since molecules were assumed to be adsorbed onto the shell surface. These results indicate that SERS signals of Raman chemicals located on the silica surface of SiO_2_@Ag@SiO_2_ NPs can be enhanced compared to the same sized Ag NP monomer regardless of binding properties to the noble metal surface.

### Detecting aniline in river water and tap water using SiO_2_@Ag@SiO_2_ NPs

**Fig 6:**
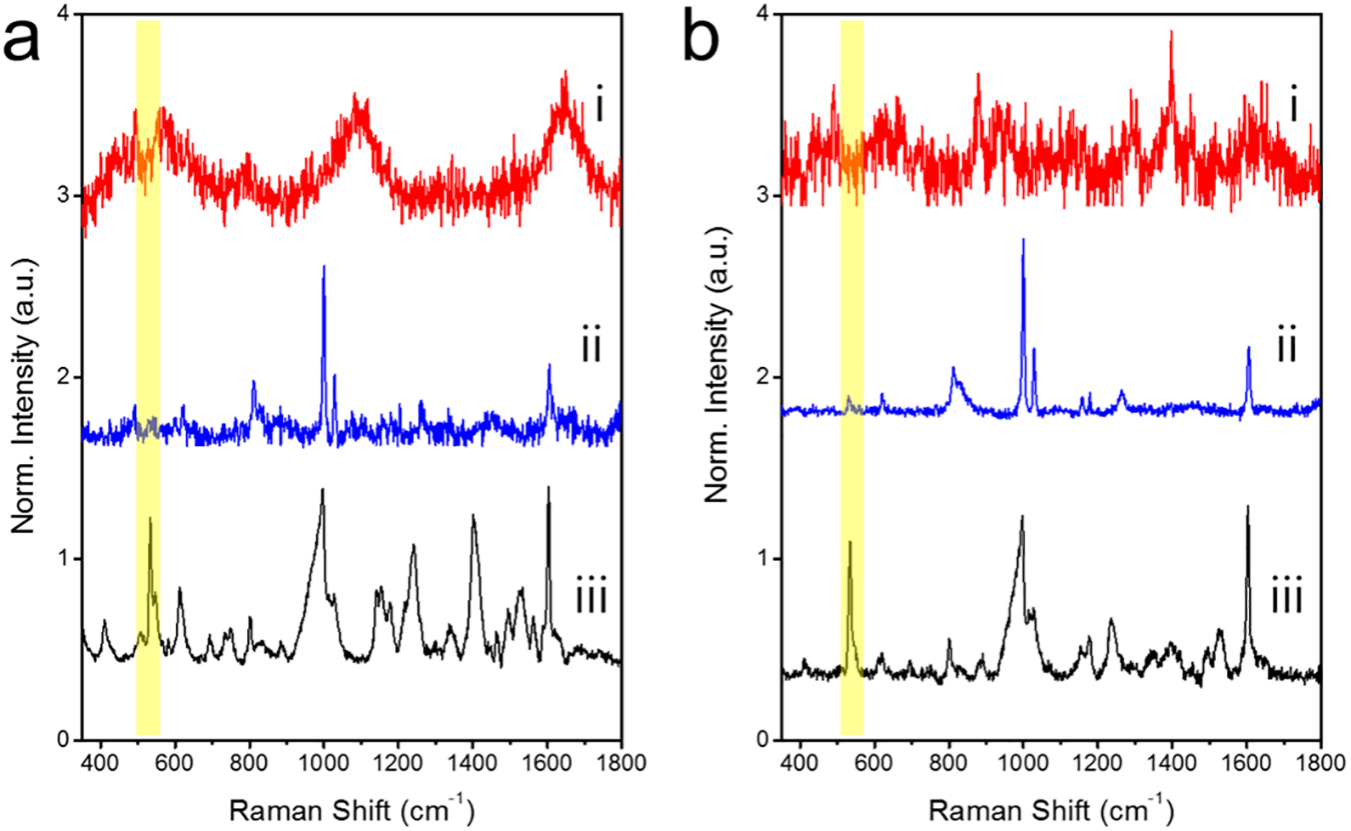
Comparison of surface-enhanced Raman scattering (SERS) spectra. (a) Raman spectra of river water. (i) River water only, (ii) aniline in river water with SiO_2_@Ag NPs, and (iii) aniline in river water with SiO_2_@Ag@SiO_2_ NPs. (b) Raman spectra of tap water. (i) Tap water only, (ii) aniline in tap water with SiO_2_@Ag NPs, (iii) aniline in tap water with SiO_2_@Ag@SiO_2_ NPs. All Raman spectra were measured at laser power of 10 mW with acquisition time of 10 s. Intensities were normalized to Raman intensity of ethanol peak at 882 cm^−1^.

The efficacy of the SiO_2_@Ag@SiO_2_ NP method for detecting aniline in river or tap water samples was evaluated. Han River (South Korea) or tap water containing 1 drop of 100 mM aniline was aliquoted into 42 μm^3^ capillary tube which was calculated from the laser beam diameter of a Raman microscope system (×10 objective lens). The tube was filled with solvent. Approximately 0.5 mL of 1 mg/mL ethanol suspension containing the silica coated assembled Ag NPs was then added into the tube to allow interaction with aniline. SERS spectra were obtained using a micro Raman system. Fig 6A shows Raman spectra of the river water (i), river water containing aniline without silica coated assembled Ag NPs (ii), and river water containing aniline with silica coated assembled Ag NPs (iii). Only the mixture of river water and silica coated assembled Ag NPs exhibited a strong aniline peak. Aniline in the river water sample was detectable based on a 520 cm^−1^ peak. Fig 6B shows Raman spectra of tap water (i), tap water containing aniline without silica coated assembled Ag NPs (ii), and tap water containing aniline with silica coated assembled Ag NPs (iii). Tap water by itself did not contain an aniline signal which was evident as a Raman shift in the 520 cm^−1^ peak. Only tap water with aniline and silica coated assembled Ag NPs exhibited a strong aniline peak (Fig 6Biii).

## Conclusion

In conclusion, thin silica shell coated Ag NPs assembled on silica NPs were fabricated in this study to detect aniline. Silica shell of SiO_2_@Ag@SiO_2_ NPs was finely controlled at a thickness of 4–5 nm. These SiO_2_@Ag@SiO_2_ NPs could detect SERS of aniline/4-ATP mixture, indicating that they could be used to detect non-thiol moiety within a thiol-based molecular mixture. LODs of these SiO_2_@Ag@SiO_2_ NPs for aniline and 4-ATP were 93 ppm and 54 ppb, respectively. E-field distribution of these assembled Ag NPs encapsulated in a silica shell was calculated by DDA. Such SiO_2_@Ag@SiO_2_ NP-based aniline detection system was successfully applied to river water and tap water. SiO_2_@Ag@SiO_2_ NP-based detection is a simple practical tool that expands the flexibility of SERS for useful applications in material science and life science. It can also be used for detection chemicals associated with food safety, drugs, explosives, and environmental pollutants.

## Supporting information

S1 Fig Surface property of oleic acid-treated SiO_2_@Ag NPs. (a) Ethylene glycol (upper layer) separated from the Ag NPs suspension; (b) oleic acid treated NPs (upper layer) separated from ethylene glycol; (c) dispersion of SiO_2_@Ag@SiO_2_ NPs in EtOH.
(TIF)

S2 Fig **Detection limit of SERS spectra of (a) aniline (at 550 cm**^**−1**^**) and (b) 4-ATP (at 1,390 cm**^**−1**^**) using SiO**_**2**_**@Ag@SiO**_**2**_ **NPs.** All spectra were measured by 532 nm photoexcitation of 10 mW laser power and 10 s acquisition. The aniline peaks were normalized to the ethanol peak at 882 cm^−1^. The pH of 4-ATP sample solution was maintained at 8.
(TIF)

S3 Fig **Raman spectra of (a) Han river and (b) tap water.** Both spectra were measured by 532 nm photoexcitation of 10 mW laser power and 10 s acquisition.
(TIF)

S4 Fig Theoretical E-field distribution of SiO_2_@Ag NPs. (a-j) E-field distribution of the SiO_2_@Ag NPs dimer with different inter-particle distances from 17–26 nm of center-to-center distances and (k) E-field distribution of SiO_2_@Ag NP monomer.
(TIF)
