# Supplementary figures and images for: Thin silica shell coated Ag assembled nanostructures for expanding generality of SERS analytes
Source: PLoS One. 2017 Jun 1;12(6):e0178651. doi: 10.1371/journal.pone.0178651 (PMC5453564; doi:10.1371/journal.pone.0178651)

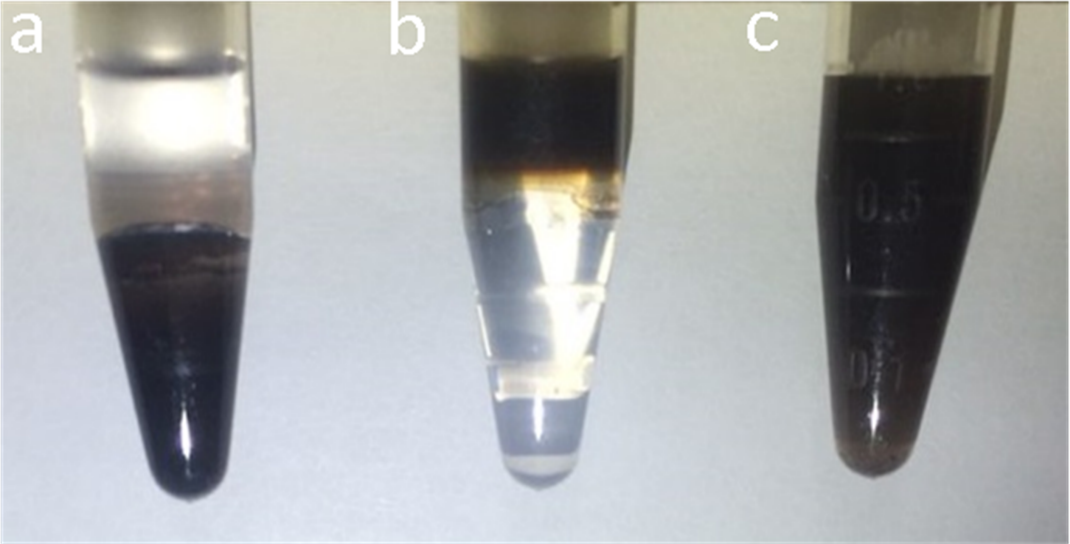

Supplement: S1 Fig — (a) Ethylene glycol (upper layer) separated from the Ag NPs suspension; (b) oleic acid treated NPs (upper layer) separated from ethylene glycol; (c) dispersion of SiO2@Ag@SiO2 NPs in EtOH. (TIF) [file pone.0178651.s001.tif]

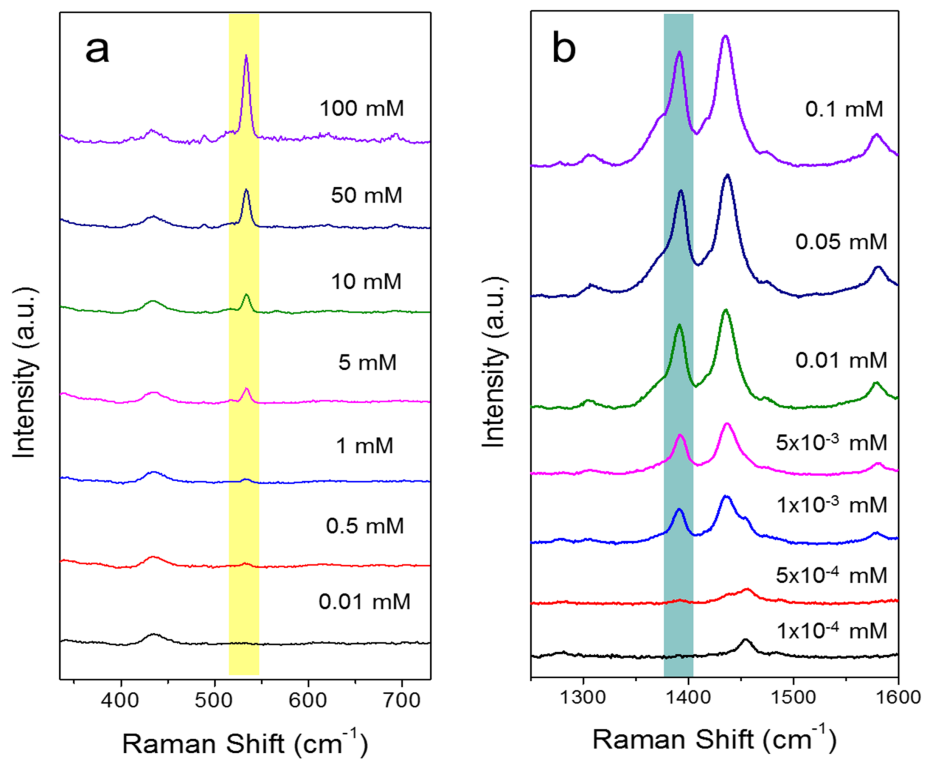

Supplement: S2 Fig — Detection limit of SERS spectra of (a) aniline (at 550 cm−1) and (b) 4-ATP (at 1,390 cm−1) using SiO2@Ag@SiO2 NPs. All spectra were measured by 532 nm photoexcitation of 10 mW laser power and 10 s acquisition. The aniline peaks were normalized to the ethanol peak at 882 cm−1. The pH of 4-ATP sample solution was maintained at 8. (TIF) [file pone.0178651.s002.tif]

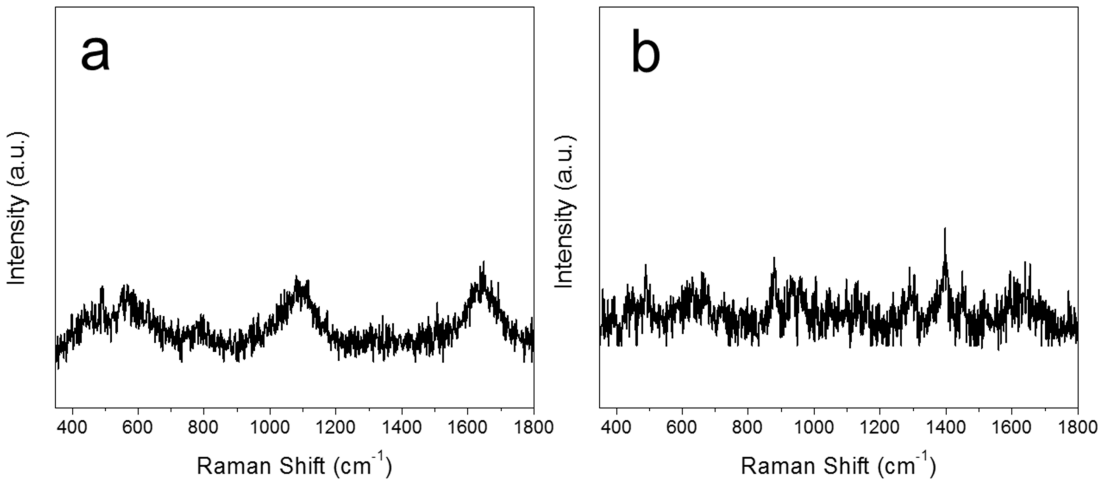

Supplement: S3 Fig — Raman spectra of (a) Han river and (b) tap water. Both spectra were measured by 532 nm photoexcitation of 10 mW laser power and 10 s acquisition. (TIF) [file pone.0178651.s003.tif]

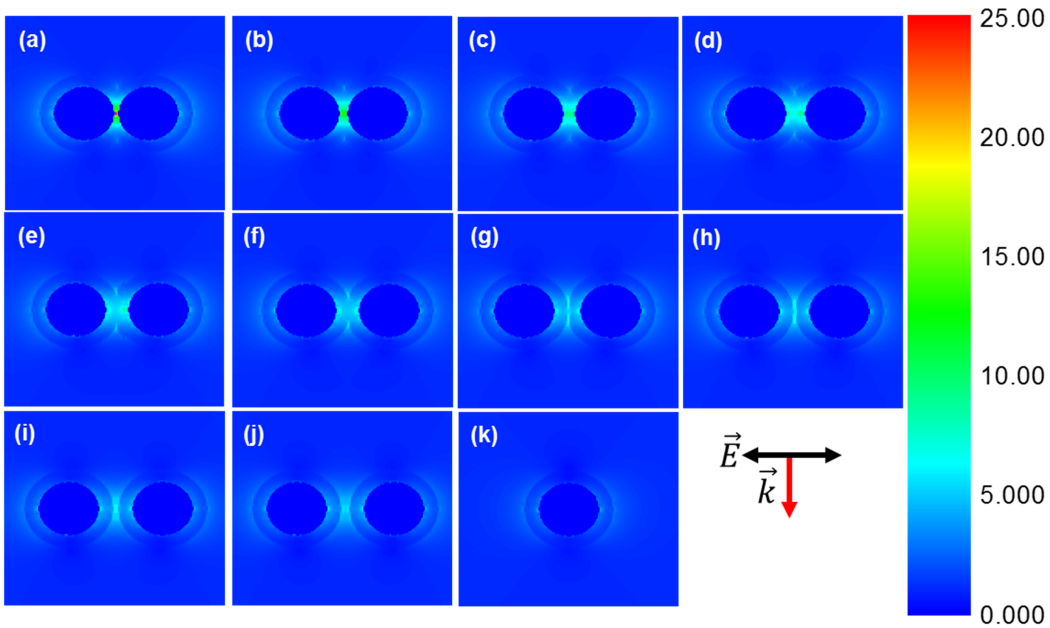

Supplement: S4 Fig — (a-j) E-field distribution of the SiO2@Ag NPs dimer with different inter-particle distances from 17–26 nm of center-to-center distances and (k) E-field distribution of SiO2@Ag NP monomer. (TIF) [file pone.0178651.s004.tif]
